# Effects of controlled hypoxemia or hypovolemia on global and intestinal oxygenation and perfusion in isoflurane anesthetized horses receiving an alpha-2-agonist infusion

**DOI:** 10.1186/s12917-017-1265-3

**Published:** 2017-11-28

**Authors:** Klaus Hopster, Liza Wittenberg-Voges, Florian Geburek, Charlotte Hopster-Iversen, Sabine B. R. Kästner

**Affiliations:** 1 Equine Clinic, University of Veterinary Medicine Hanover, Foundation, Bünteweg 9, D-30559 Hanover, Germany; 2 Department of Clinical Studies-NBC, School of Veterinary Medicine, University of Pennsylvania, 382 West Street Road, Kennett Square, PA 19348 USA

**Keywords:** Gastrointestinal tract, Hypoxemia, Hypovolemia, Laser Doppler flowmetry, Microperfusion

## Abstract

**Background:**

Aim of this prospective experimental study was to assess effects of systemic hypoxemia and hypovolemia on global and gastrointestinal oxygenation and perfusion in anesthetized horses. Therefore, we anesthetized twelve systemically healthy warmblood horses using either xylazine or dexmedetomidine for premedication and midazolam and ketamine for induction. Anesthesia was maintained using isoflurane in oxygen with either xylazine or dexmedetomidine and horses were ventilated to normocapnia. During part A arterial oxygen saturation (SaO_2_) was reduced by reducing inspiratory oxygen fraction in steps of 5%. In part B hypovolemia was induced by controlled arterial exsanguination via roller pump (rate: 38 ml/kg/h). Mean arterial blood pressure (MAP), heart rate, pulmonary artery pressure, arterial and central venous blood gases and cardiac output were measured, cardiac index (CI) was calculated. Intestinal microperfusion and oxygenation were measured using laser Doppler flowmetry and white-light spectrophotometry. Surface probes were placed via median laparotomy on the stomach, jejunum and colon.

**Results:**

Part A: Reduction in arterial oxygenation resulted in a sigmoid decrease in central venous oxygen partial pressure. At SaO_2_ < 80% no further decrease in central venous oxygen partial pressure occurred. Intestinal oxygenation remained unchanged until SaO_2_ of 80% and then decreased. Heart rate and pulmonary artery pressure increased significantly during hypoxemia. Part B: Progressive reduction in circulating blood volume resulted in a linear decrease in MAP and CI. Intestinal perfusion was preserved until blood loss resulted in MAP and CI lower 51 ± 5 mmHg and 40 ± 3 mL/kg/min, respectively, and then decreased rapidly.

**Conclusions:**

Under isoflurane, intestinal tissue oxygenation remained at baseline when arterial oxygenation exceeded 80% and intestinal perfusion remained at baseline when MAP exceeded 51 mmHg and CI exceeded 40 mL/kg/min in this group of horses.

**Trial registry number:**

33.14-42,502-04-14/1547.

## Background

Global oxygen delivery (ḊO_2_) is determined by total blood flow or cardiac output, and arterial oxygen content, which depends on hemoglobin concentration and its saturation with oxygen as well as the arterial oxygen partial pressure (PaO_2_). Impaired ḊO_2_ to the periphery might contribute to the high prevalence of equine fatalities directly related to anesthesia and/or surgery [1, 2] by drug induced reduction of cardiovascular function, hemorrhage and arterial hypoxemia.

During general anesthesia, particularly if in dorsal recumbency, horses may develop a large alveolo- arterial oxygen partial pressure gradient and become hypoxaemic [3]. Hypoxemia (defined as an arterial partial pressure of oxygen <60 mmHg) leading to inadequate ḊO_2_ to peripheral tissues during anesthesia would seem a potential cause of increased mortality [4] and an impairment of splanchnic oxygenation can contribute to alterations in intestinal integrity and to postoperative colic. During severe hypoxemia ḊO_2_ may become diminished so that oxygen consumption (VO_2_) becomes linearly dependent on ḊO_2_. As a linear relationship develops between both, ḊO_2_becomes inadequate to maintain aerobic metabolism leading to tissue hypoxia and necrosis. To our knowledge there are no studies investigating effects of arterial hypoxemia on regional gastrointestinal oxygenation in horses.

Dose-dependent cardiovascular depression by isoflurane has been well described and decreased cardiac index (CI) is a direct effect of isoflurane on myocardial contractility, leading to a reduction in stroke volume and consequent, in combination with its vasodilatory effects, to a reduction in mean arterial blood pressures (MAP) [5]. In horses it has been shown, that states of low blood pressure and low cardiac output will lead to impaired intestinal perfusion [6]. However, information on the influence of acute blood loss on gastrointestinal microperfusion is limited in this species. In man, severe hemorrhage is associated with redistribution of cardiac output to vital organs (brain, heart) but reduced perfusion and oxygen delivery to others, such as the gut [7]. Impairment of splanchnic perfusion and/or oxygenation can contribute to alterations in intestinal motility [8] and breach of the intestinal mucosal barrier [9] leading to septicemia and ileus. In horses, studies investigating effects of acute blood loss on global and intestinal perfusion during anesthesia are missing.

Aim of this study was to evaluate effects of hypoxemia and hypovolemia on global perfusion and oxygenation and microperfusion and oxygenation of the gastrointestinal tract in anesthetized horses using surface lightguide tissue spectrophotometry combined with laser Doppler flowmetry.

## Results

### Part A: Hypoxemia

There were no differences between horses receiving either dexmedetomidine or xylazine. At the beginning of the experiment all horses were sufficient oxygenated with an arterial oxygen saturation values (SaO_2_) > 95% and PaO_2_ ranging between 82 mmHg and 389 mmHg. Due to the high variation in lung function at the beginning of the experiment, inspiratory oxygen concentrations resulted in different SaO_2_ during the down titration of FiO_2_.

**Fig. 1:**
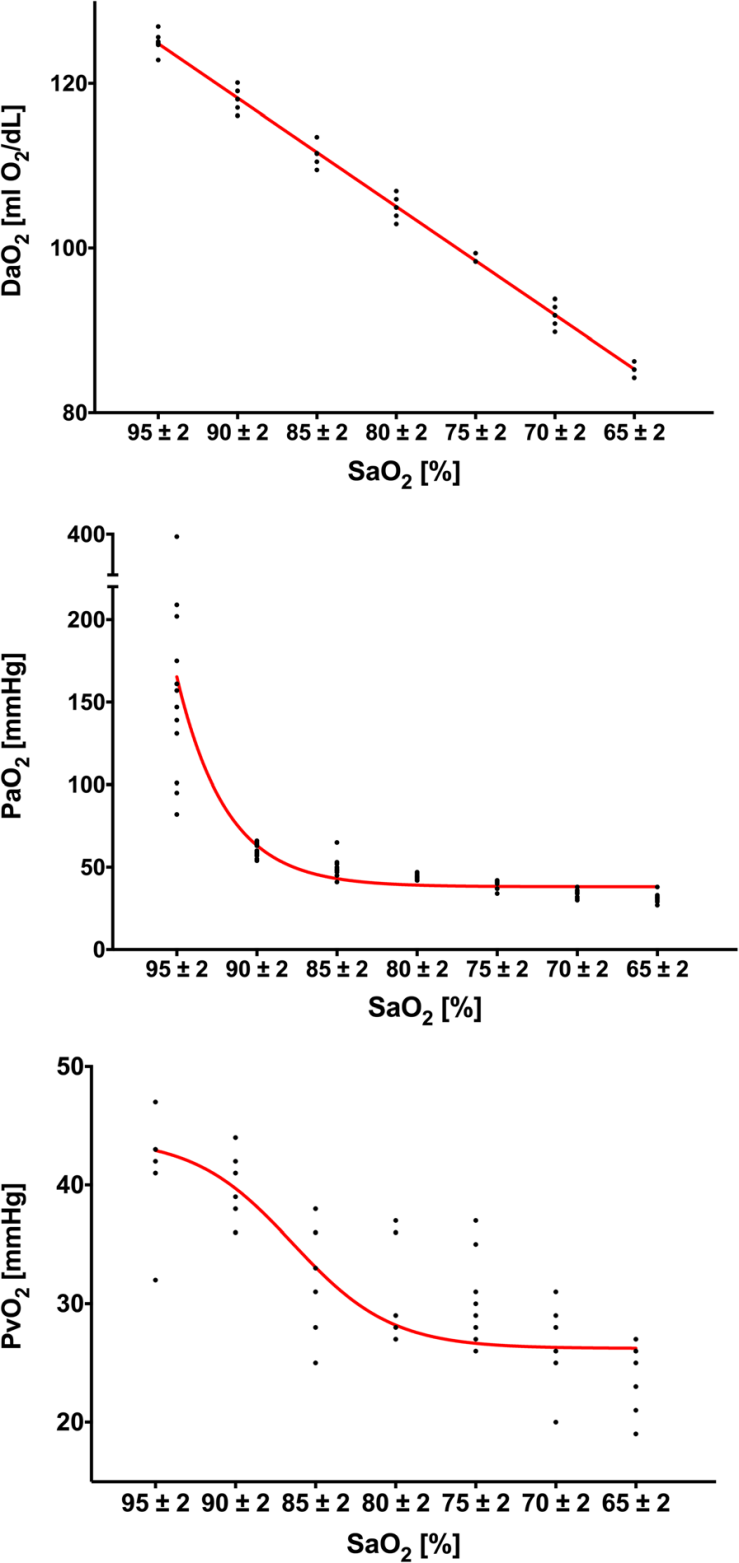
Oxygen delivery (ḊO_2_), arterial oxygen partial pressure (PaO_2_) and mixed venous oxygen partial pressure (PV̄̀O_2_) at different arterial oxygen saturation values (SaO_2_) in anaesthetized horses during controlled hypoxemia

**Fig. 2:**
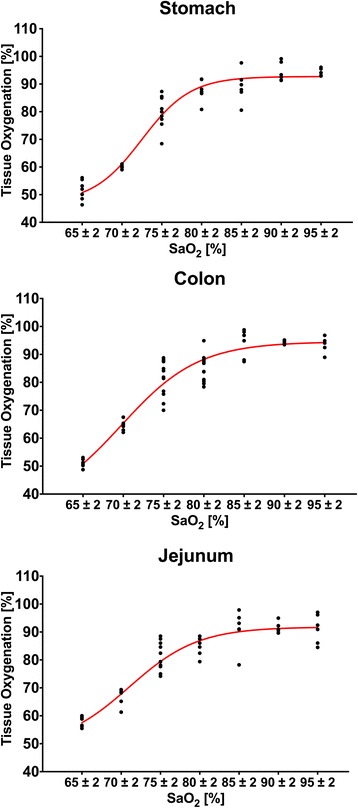
Non-linear relation between tissue oxygenation of the stomach (gastric oxygenation), the jejunum (jejunal oxygenation) and the colon (colonic oxygenation) and the arterial oxygen saturation (SaO_2_)

Reducing the inspiratory oxygen concentration resulted in a decrease in ḊO_2_, PaO_2_ and mixed venous oxygen partial pressure (PV̄̀O_2_) in all horses (Fig. 1). As expected by the shape of the oxygen dissociation curve relation between decrease in SaO_2_ and PaO_2_ was not linear. The reduction in SaO_2_ resulted in a non-linear decrease in intestinal tissue oxygenation (Fig. 2) and in PV̄̀O_2_ (Fig. 1). MAP and CI remained stable but heart rate increased significantly when SaO_2_ decreased below 72% (Table 1). There was a constant, but non-significant increase in pulmonary artery pressure (PAP) (Table 1). Gastrointestinal tissue oxygenation (sO_2_) decreased rapidly and significantly when SaO_2_ dropped below values 80 ± 2% (Fig. 2).

**Table 1:** Mean and standard deviation of cardiac index (CI), mean arterial blood pressure (MAP), heart rate (HR) and pulmonary artery pressure (PAP), central venous oxygen saturation (SV̄̀O_2_) and oxygen extraction ratio (O_2_ER) at different arterial oxygen saturation values (SaO_2_) in anesthetized horses during controlled hypoxemia

	SaO_2_ (%)						
	95 ± 2	90 ± 2	85 ± 2	80 ± 2	75 ± 2	70 ± 2	65 ± 2
CI (ml/kg/min)	61 ± 16	62 ± 15	63 ± 16	61 ± 11	67 ± 14	73 ± 18	75 ± 13 ^a^
MAP (mmHg)	72 ± 9	75 ± 8	76 ± 12	77 ± 11	76 ± 10	75 ± 11	78 ± 9
HR (beats/min)	30 ± 2	30 ± 3	31 ± 5	31 ± 4	33 ± 6	37 ± 6 ^a^	42 ± 8 ^a^
PAP (mmHg)	16 ± 4	18 ± 3	20 ± 3	21 ± 4	22 ± 4	23 ± 5	25 ± 6
SV̄̀O_2_ (%)	74 ± 2	67 ± 4	61 ± 2 ^a^	57 ± 3 ^a^	55 ± 3 ^a^	53 ± 2 ^a^	49 ± 4 ^a^
O_2_ER (%)	21 ± 2	23 ± 2	26 ± 2 ^a^	29 ± 3 ^a^	25 ± 3	25 ± 2	22 ± 4

^a^Statistically significant different from baseline (SaO_2_ 95%)

### Part B: Hypovolemia

**Fig. 3:**
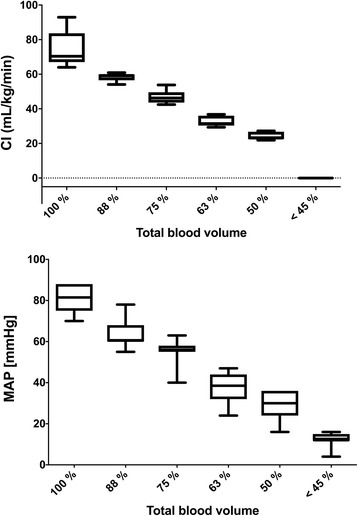
Box and whiskers plots of cardiac index (CI) and mean arterial blood pressure (MAP) during controlled exsanguination at different states of total blood loss; the boxes are indicating the 75% quantile, the whiskers min to max and the bar the mean value

**Fig. 4:**
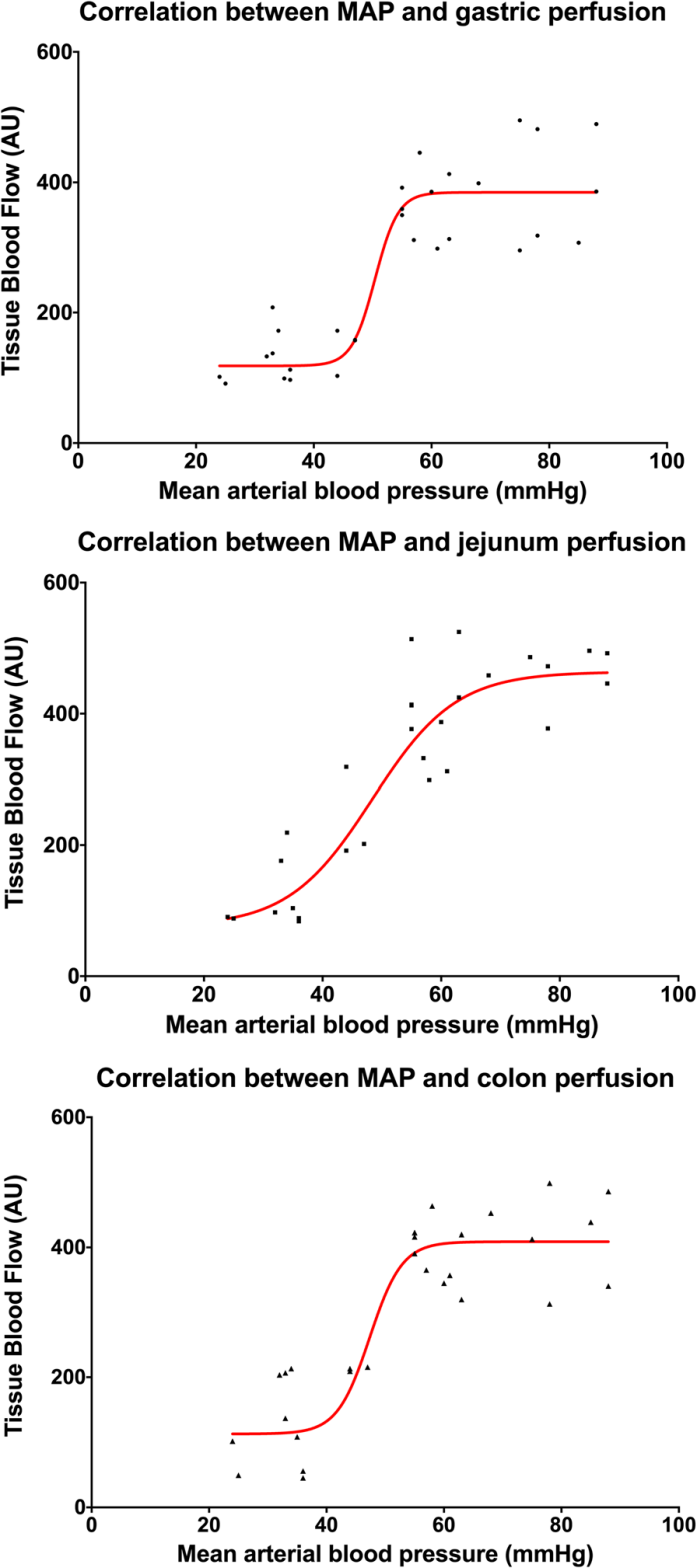
Non-linear relation between mean perfusion of the stomach (total gastric blood flow), the jejunum (total jejunal blood flow) and the colon (total colonic blood flow) and mean values of mean arterial blood pressure (MAP) during controlled exsanguination in anaesthetized horses

**Fig. 5:**
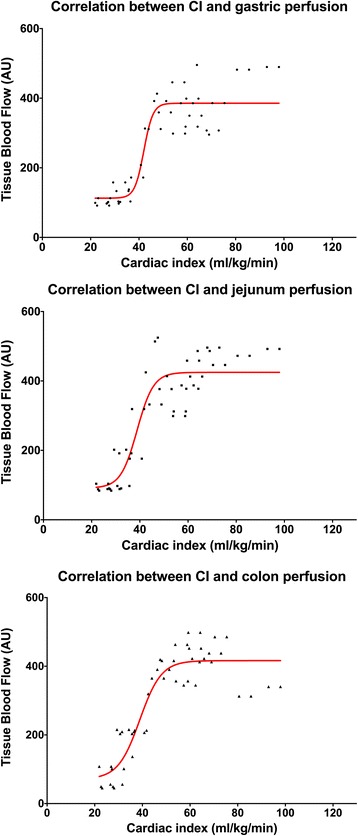
Non-linear relation between mean perfusion of the stomach (total gastric blood flow), the jejunum (total jejunal blood flow) and the colon (total colonic blood flow) and mean values of the cardiac index (CI) during controlled exsanguination in anaesthetized horses

Measurements during exsanguination were performed at baseline blood volume and at 88%, 75%, 63%, 50% and less than 45% of that value. All horses died after a total blood loss of 55 to 62% (no pulse and no cardiac output detectable). Exsanguination resulted in a continues decrease in CI and MAP (Fig. 3). Intestinal perfusion remained stable for the first 3 measurements; at a total blood volume of less than 63% (CI and MAP lower 40 ± 3 mL/kg/min and 51 ± 5 mmHg, respectively) a significant decrease of tissue blood flow of stomach, jejunum and colon occurred (Figs. 4 and 5). No significant differences were found between horses receiving either dexmedetomidine or xylazine.

## Discussion

In horses, especially in non-healthy colic patients, adequate oxygenation is regularly a problem [10]. One aim of this study was to evaluate effects of severe hypoxemia during anesthesia on gastrointestinal oxygenation in horses. Reducing inspiratory oxygen fraction resulted in a decrease in PaO_2_ and SaO_2_ and therefore in a decrease in arterial oxygen content (CaO_2_) and ḊO_2_, as CI remained constant during FiO_2_ reduction. Under physiologic steady-state conditions, intestinal VO_2_ is independent of ḊO_2_ and determined by cellular metabolic needs [11–13]. A moderate decrease in ḊO_2_ is compensated by an increase in oxygen extraction by the tissue [14]. In our study, a decrease in SaO_2_ < 90% resulted in a decrease in SvO_2_ and PvO_2_. At this point the oxygen extraction ratio increased as well and therefore maintained VO_2_ and oxygenation of the tissue. When ḊO_2_ is severely compromised in situations of severe hypoxia or ischemia, VO_2_ becomes limited by and therefore dependent on ḊO_2_ [15, 16]. Intestinal oxygenation was initially stable when starting the reduction of FiO_2_ and SaO_2_ and therefore reducing ḊO_2_, but decreased rapidly when SaO_2_ dropped below values of less than 80%, and the compensatory increase in oxygen extraction reached the limit. During progressive hypoxia in resting dogs, ḊO_2_ needed to decrease below 50% of baseline before VO_2_ and jejunal tissue oxygenation decreased [14, 17]. In other animals (rats, pigs, lambs) critical values also ranged between 50 and 60% [15, 18, 19] whereas in our study a ḊO_2_ reduction of more than 30% resulted in a decrease in tissue oxygenation. Intestinal tissue oxygenation is relatively resistant to hypoxemia as compared to ischemia and the capacity to tolerate pure hypoxemia is dependent on perfusion being preserved to intestinal tissue [17]. All previous measurements were either performed in isolated organs or in previous equipped and awake animals. Our horses were anesthetized and therefore, had per se a reduced cardiovascular function (lower CI and MAP) but also reduced oxygen requirements compared to standing and resting animals.

Decreasing ḊO_2_ resulted in a significant increase in heart rate but blood pressure and CI did not change. The increase in heart rate can be explained by an increase in sympathetic tone by hypoxemia [20]. Despite an increase in sympathetic nerve activity, acute systemic hypoxemia leads to decreased blood pressure due to vasodilation [21]. In contrast to these findings blood pressure remained stable in our study which can be explained by the use of constant rate infusion of alpha-2-agonists during anesthesia masking/reducing vasodilative effects of hypoxia by their vasoconstrictive properties [22]. Under hypoxic conditions a depression of the myocardium is observed resulting in a reduction of myocardial performance and cardiac ejection fraction [23, 24]. The fact that there were no significant changes in cardiac index can be explained by a partial compensation of the reduced myocardial performance by the sympathetic-mediated increase of the heart rate. Further the time of hypoxemia was probably too short to cause severe myocardial changes. The fact that we had no changes in either CI or MAP further might explain the lack in changes in gastrointestinal perfusion during hypoxemia in this study [6]. We cannot exclude that longer or more severe periods of hypoxemia may affect the gastrointestinal perfusion in horses.

The second part of this study was performed to investigate effects of a hemorrhagic shock on central and intestinal perfusion in anesthetized horses. All animals responded very similar to the acute blood loss. There was a close inverse and linear correlation of arterial blood pressure and cardiac index with the amount of blood loss as indices of global perfusion. The pathophysiological response to hemorrhagic shock is characterized by vasoconstriction with an initial increase in MAP followed by a marked decrease in MAP and CI when acute loss of 20% of the total blood volume is exceeded [25–27]. In the presence of cardiovascular diseases, anemia or otherwise compromised cardiovascular function or by using different drugs as alpha-2-agonists these responses can vary.

In our horses a drop in MAP and CI was already apparent when total blood loss exceeded 10%. A study in horses anesthetized with halothane or isoflurane showed that severe hemorrhage leads to a significant drop in blood pressure that is most prominent in the initial phase [28] and less linear than observed in non-anesthetized horses. This can be explained by the influence of isoflurane leading to a reduction in stroke volume as well as causing vasodilation [5]. In our study heart rate remained stable until blood loss exceeded 25%. This is also consistent with results decribed by Wilson et al. [28]. Other studies describe an increase in heart rate with progressive hypovolemia. Often, tachycardia is one of the first clinical signs with blood loss [29–31], but in human medicine between 7% and 28% of patients in hypovolemic shock are presented with bradycardia [32, 33]. Common causes for bradycardia are drugs or an increase in the parasympathetic drive. An explanation for the delayed tachycardia might be the presence of high doses alpha-2-agonists in our horses leading to a high parasympathic tone or the isoflurane-induced depression of the sympathetic tone. Further this response could also vary between species.

The perfusion of the intestinal organs remained unchanged until a total blood loss of 37% of the calculated total blood volume resulted in cardiac index and blood pressure values of less than 40 ± 3 ml/kg/min and 51 ± 5 mmHg, respectively. In dogs, pigs and rats it has been shown that in a hypovolemic situation the perfusion of the skin, the skeletal muscles and the gastrointestinal tract is not a priority and is disproportionally reduced compared to the muscle tissue [34–36]. In contrast a study with nine healthy horses under isoflurane anesthesia showed that gastrointestinal perfusion was preserved until a threshold blood pressure of about 60 mmHg or cardiac output lower 50 mL/kg /min were reached [6]. These authors assumed that differences in the regulation of large and small vessel tone [37]. As well as differences between species could be the reason for this. Therefore, it is possible that in our horses despite this reduced blood flow in the larger vessels the local microperfusion of the gastrointestinal perfusion is maintained for a wider range. We further claim that in horses under general anesthesia the intestinal perfusion is sustained for a longer period of acute blood loss.

One limitation of the method we used for measuring the perfusion, the laser Doppler flowmetry, is that this method only allows measurements in arbitrary units and not in quantifiable units. This is because calibration of the output voltage of a laser Doppler flowmeter is difficult, due to the small sample volume size and variations in the optical properties of the tissues [38]. Therefore, interpreting the horses local blood flow technically is only possible in comparison to baseline values, which is one of the reasons for the numerous baseline measurements in between each drug in this experiment. Comparing the results directly to other studies is thereby limited to comparing a trend rather than actual numerical data. Further the used device is calibrated for human blood characteristics, and it is possible that absolute values measured by the O2C were different from real tissue oxygenation. Nonetheless, changes in oxygenation measured in our study were considered reliable because all measurements were performed over time with the same probe in the same animal. Another limitation is that we used a balanced anesthesia protocol with Isoflurane in combination with an alpha-2-agonist. The use of vasoactive drugs including during the anesthetic maintenance phase might have an influence on perfusion parameters and the response of the horses to hypoxemia and exsanguination. We decided to evaluate these effects on a clinical acceptable anesthesia protocol. Inhalational agents like isoflurane are accompanied by dose-related cardiovascular depression affecting cardiac output, blood pressures and muscle perfusion and therefore often combined with alpha-2-agonists like xylazine or dexmedetomidine due to their great potential to reduce the MAC of inhalation agents. However, the lack of a control group not receiving alpha-2-agonists makes interpretation of this difficult. All horses were part of another experiment making the use of two different alpha-2-agonists necessary. Both drugs were used in equipotent doses [39] and infusion time was long enough to assume a steady state.

Another point that needs to be addressed is anesthetic preconditioning, a mechanism referring to changes on a biomolecular level to improve tissue tolerance for hypoxemic conditions. It has been shown that isoflurane as well as different alpha-2-agonists can lead to drug induced preconditioning [40, 41]. Whether this had contributed to our results and to which amount cannot be clarified with our results.

Although there is clear evidence that hemodynamic parameters are stable and time-independent during isoflurane anesthesia in horses [5, 6], it is possible that the hypovolemia experiment was effect first by the long duration of anesthesia and second by the hypoxemia experiment. Due to ethical reasons, we decided to use these horses for multiple experiments as euthanasia was necessary in these animals for tissue sampling of an associated surgical study.

## Conclusion

Under isoflurane anesthesia in combination with an alpha-2-agonist constant rate infusion the intestinal tissue compensates a decrease in ḊO_2_ initially by an increase in oxygen extraction but becomes dependent on ḊO_2_ during severe hypoxemia with SaO_2_ < 80%.

During acute blood loss in anesthesia intestinal perfusion seems to be preserved until a threshold blood pressure (< 50 mmHg) or CI (< 40 mL/kg/min) is reached. With high doses of alpha-2-agonists heart rate might not be a good predictor of hemorrhage in the early state of blood loss. Maintaining perfusion pressure is important for intestinal perfusion.

## Methods

### Animals

The study was approved by the Ethics Committee for Animal Experiments of Lower Saxony, Germany, number 33.14-42,502-04- 14/1547. Twelve experimental horses (six mares, three geldings and three stallions) with a body weight of 540 ± 41 kg (mean ± SD) and an age of 7 ± 6 years were used for this study. All horses were systemically healthy based on physical examination and routine haematological and biochemical blood work. They were part of an additional anesthesia study and a terminal, experimental surgery study and were euthanized for tissue sampling at the end of anesthesia using pentobarbital (60 mg/kg i.v.).

### Anesthesia

Horses were sedated with xylazine (0.5 mg/kg, Xylavet, CP-Pharma, Burgdorf, Germany) or dexmedetomidine (3.5 μg/kg, Dexdomitor, Pfizer Tiergesundheit GmbH, Germany). Induction of anesthesia with midazolam (0.05 mg/kg, Midazolam-ratiopharm, ratiopharm, Ulm, Germany) and ketamine (2.2 mg/kg, Narketan, Vetoquinol, Ravensburg, Germany) was identical in all horses. Anesthesia was maintained with isoflurane (IsofluranCP, CP-Pharma, Burgdorf, Germany) in pure oxygen in combination with a constant rate infusion of 1 mg/kg/h xylazine or 7 μg/kg/h dexmedetomidine. The end tidal isoflurane concentration was maintained between 1.1 and 1.2 Vol.% and kept constant during the experimental procedure and horses received lactated Ringer’s solution (B. Braun, Melsungen, Germany) at a rate of 10 ml/kg/h. After induction and intubation horses were positioned on a surgical table in dorsal recumbency. Controlled mechanical ventilation was performed with a pressure cycled large animal ventilator (Model JAVC 2000 J.D. Medical Distributing Company Phoenix, USA) using intermittent positive pressure ventilation with an inspiratory pressure of 25 cm H_2_O. Respiratory rate was adjusted to maintain arterial partial carbon dioxide pressure (PaCO_2_) between 40 and 45 mmHg.

### Instrumentation

Before anesthesia the skin over the right and left jugular vein was clipped and subcutaneously infiltrated with mepivacaine (Scandicain 2%, AstraZeneca GmbH, Germany). One 12 G catheter (EquiCathTM Fastflow®, Brau, Melsungen, Germany) was placed into the left jugular veins and two 8F catheter (BD CritiCath; BD Critical Care Systems, USA) introducers in the right jugular vein to facilitate the placement of two balloon tipped catheters. A Swan-Ganz standard thermodilution pulmonary artery catheter (BD CritiCath; BD Critical Care Systems, USA) with a length a 110 cm was placed into the pulmonary artery and a second balloon tipped catheter (Arrow 5 Fr 110 cm Balloon Wedge Pressure Catheter, Teleflex, Germany) was placed into the right atrium. Correct placement was confirmed by visual inspection of the pressure waveforms and by transthoracic ultrasonography.

During anesthesia, the transverse facial artery was cannulated with a 20 G catheter (VenocanTM IV Catheter, Kruuse, Langeskov, Denmark) for invasive blood pressure monitoring and arterial blood sampling. The catheters were connected to calibrated pressure transducers (Gould Statham Transducer, PD 23 ID, USA) via fluid-filled extension lines. The pressure transducers were positioned at the level of the sternal manubrium. A combined spectrophotometry and laser-doppler flow probe of the micro-lightguide spectrophotometer O2C (Oxygen to See, LEA Medizintechnik) was placed via median laparotomy on the serosal surface of the stomach, the jejunum and the pelvic flexion of the colon.

### Measured variables

Recording and evaluation of the data started 180 min after induction of anesthesia and after finishing another conducted study. MAP, PAP, heart rate (HR), respiratory rate, end tidal isoflurane concentration (E_T_Iso) and FiO_2_ were measured continuously with a standard anesthesia monitor^o^ and recorded.

For cardiac output measurements, the bolus thermodilution (BTD) technique was used. Therefore iced 5% dextrose solution (one mL per 15 kg bodyweight) was injected into the right atrium and the temperature change was measured via an inline temperature probe positioned in the pulmonary artery. Five injections were performed and the average of the closest three values was used. The CO was measured and the CI was calculated.

Arterial and mixed venous blood samples were taken and arterial pH, PaO_2_, PV̄̀O_2_ and PaCO_2_ as well as arterial and mixed venous hemoglobin concentrations and arterial and mixed venous oxygen saturation (SaO_2_, SV̄̀O_2_) and measured immediately after sampling (AVL995, AVL Medizintechnik, Germany).

Oxygen delivery to the tissue was calculated using the standard formula:

$$ {\dot{\mathrm{D}} \mathrm{O}}_2=\mathrm{CO}\times \left(1.34\times \left[\mathrm{hemoglobin}\  \mathrm{concentration}\right]\times {\mathrm{SaO}}_2\right)+\left(0.0031\times {\mathrm{PaO}}_2\right) $$

Oxygen extraction ratio was calculated using the standard formula:

$$ {\mathrm{O}}_2\mathrm{ER}=\left[\left({\mathrm{CaO}}_2-\mathrm{CV}\bar \grave {\mathrm{O}}_2\right)/{\mathrm{CaO}}_2\right]\times 100\% $$

With CaO_2_ = 1.34 × [hemoglobin concentration] × SaO_2_) + (0.0031 × PaO_2_).

And with CV̄̀O_2_ = 1.34 × [hemoglobin concentration] × SV̄̀O_2_) + (0.0031 × PV̄̀O_2_)

### Tissue oxygenation and blood flow

Gastrointestinal tissue oxygenation (sO_2_ in %) and blood flow (flow) were measured by the micro-lightguide spectrophotometer O2C as described previously [42]. This device uses the laser Doppler shift to measure tissue blood flow and white light spectroscopy for measuring the tissue oxygenation (sO_2_). A probe with a penetration depth of 2.5 mm was used for all measurements. The surface of this probe was placed on the mucosa of the stomach, the jejunem (about 3 m orally from the ileum) and the pelvic flexure of the large colon. Flow and saturation were recorded for at least 30 s at every measuring time point. Before each recording, quality of the laser Doppler signal was evaluated on a monitor so that identification of incorrect probe positioning or movement artefacts was possible.

### Experimental protocol

#### Part A: Hypoxemia

After equilibration and instrumentation two baseline measurements were performed at a stable plane of anesthesia with an inspiratory oxygen concentration of >95%. Thereafter FiO_2_ (constant fresh gas flow of 8 l per minute) was stepwise decreased to 75%, 55%, 40%, 30%, 20%, 15% and 10% by mixing inspiratory oxygen with nitrogen up stream of the vaporizer. Measurements were performed 10 min after reaching the new inspiratory oxygen concentration. Decreasing inspiratory oxygen concentration was terminated when a horse had SaO_2_ of 65% or less. About 10 min after reaching the targeted FiO_2_ the MAP and CI as well as the sO2 were measured and arterial and central venous blood samples were taken.

For comparison of global oxygenation (SaO_2_) and peripheral oxygenation (tissue oxygenation) SaO_2_ values were grouped as follows: 95 ± 2%, 90 ± 2%, 85 ± 2%, 80 ± 2%, 75 ± 2%, 70 ± 2% and 65 ± 2% independent from FiO_2_.

After completion of hypoxic measurements, nitrogen flow was stopped and pure oxygen was used. After 30 min inspiratory oxygen concentration was 90% or higher and no horse showed signs of hypoxemia (SaO_2_ > 95%). Gastrointestinal oxygenation recovered back to baseline values.

#### Part B: Hypovolemia

Surgical preparation of the carotid artery was performed and an 8G catheter was placed into the artery. The catheter was connected to a roller pump (IP 65, Ismatec, Germany) to ensure controlled and continuous exsanguination and Ringer-Lactate-Infusion was stopped.

Total blood volume of the horses was estimated being about 7.6% of the total body weight (bwt) or 76 mL/kg bwt [43]. After calculation total blood volume of each horse pumping rate was set to get an exsanguination rate of 50% total blood volume loss per hour (38 mL/kg bwt/h).

The HR, MAP, PAP and CI as well as the intestinal blood flow were measured every 15 min starting at baseline blood volume and at 88%, 75%, 63%, 50% and less than 45% of that value. Central and peripheral perfusion parameters measurements were continued until horses had no detectable pulse or cardiac output.

### Statistical analysis

Statistical significance was attributed when *p* < 0.05. Analyses were carried out with the statistical software SAS, version 9.1.3 (SAS Institute, Cary, NY, USA) and GraphPad Prism 5 (GraphPad Software, Inc., USA). For the analysis of the linear model, the procedure MIXED was used. The parameters sO_2_ and flow were sampled with 2 Hz. Measurements were performed over at least 25 to 30 s resulting in 50 to 60 values for each parameter and time point. The mean of these single measurements was calculated and used for this set time point. Normal distribution of model residuals of dependent variables was confirmed by Shapiro-Wilks-Test. Data is presented as mean ± standard deviation. A two way analysis of variance and Tukey’s post hoc test were used for comparing the measured parameters by period of time (repeated measurements). The non-linear curve fitting was used to construct the curve that has the best fit to the data points in Figs. 1, 2, 4 and 5.

## Abbreviations

BTD: Bolus thermodilution
CaO_2_: Arterial oxygen content
CI: Cardiac index
CO: Cardiac output
CV̄̀O_2_: Mixed venous oxygen content
CVP: Central venous pressure
ḊO_2_: Oxygen delivery
E_T_Iso: End tidal isoflurane concentration
FiO_2_: Inspiratory oxygen fraction
HR: Heart rate
IPPV: Intermittent positive pressure ventilation
MAP: Mean arterial blood pressure
O_2_ER: Oxygen extraction ratio
PaCO_2_: Arterial partial carbon dioxide pressure
PaO_2_: Arterial partial oxygen pressure
PAP: Pulmonary artery pressure
PV̄̀O_2_: Mixed venous partial oxygen pressure
SaO_2_: Arterial oxygen saturation
sO_2_: Tissue oxygenation
SV̄̀O_2_: Mixed venous oxygen saturation
VO_2_: Oxygen consumption
